# A report study of successful surgical treatment of an aorto-left ventricular tunnel in a 5-year-old boy: differential diagnosis from other congenital heart diseases with similar clinical symptoms

**DOI:** 10.1186/s43044-025-00668-6

**Published:** 2025-07-28

**Authors:** Alireza Yaghoubi Golverdi, Amirhossein Jalali, Mohammad Mahdavi, Seyed Salaheddin Nabavi, Mahmoud Ganjifard, Maryam Bahramian, Mozhgan Bahramian, Seyed Shervin Shafiei

**Affiliations:** 1 Congenital Heart Diseases Research Center, Rajaie Cardiovascular Institute, Tehran, Iran; 2 Department of General Surgery, School of Medicine, Imam Khomeini Hospital, Golestan Hospital, Ahvaz Jundishapur University of Medical Sciences, Ahvaz, Iran; 3 Department of Anesthesiology and Critical Care, Faculty of Medicine, Birjand University of Medical Sciences, Birjand, Iran; 4 School of Medicine, Birjand University of Medical Sciences, Birjand, Islamic Republic of Iran; 5 Cardiac Surgery Department, Rajaie Cardiovascular Institute, Tehran, Iran

**Keywords:** Aorto-left ventricular tunnel, Congenital heart diseases, Left ventricle

## Abstract

**Background:**

Aorto-left ventricular tunnel (ALVT) is a rare congenital anomaly. In this condition, the aorto-ventricular tunnel is a congenital extracardiac channel that connects the ascending aorta to the left ventricle above the sinotubular junction.

**Case presentation:**

A 5-year-old boy presented with fatigue during physical activity, leading to visiting a pediatric specialist. Upon examination, a continuous murmur, predominantly diastolic, was detected. Suspecting aortic insufficiency, the patient was referred for further evaluation. Transthoracic echocardiography revealed a congenital ALVT.

**Conclusion:**

Although congenital ALVT is a rare congenital heart disease, its clinical symptoms may overlap with other congenital heart diseases, such as tetralogy of Fallot without pulmonary stenosis and patent ductus arteriosus. Differential diagnosis, such as the absence of a wide pulse pressure, can be helpful in distinguishing between these conditions.

## Background

Aorto-ventricular tunnel (AVT) is a rare congenital heart defect first described in 1963 by Levy et al. The aorto-left ventricular tunnel (ALVT) is an extremely rare extracardiac congenital heart defect, with a prevalence of 1 in 1000 congenital heart diseases. To date, approximately 130 cases have been reported. In this condition, the channel is located between the ascending aorta and the left ventricle. It extends superiorly above the sinotubular junction and inferiorly into the cavity of the left ventricle. Most patients experience symptoms of heart failure within the first year of life. The onset, severity, and progression of heart failure vary, ranging from intrauterine fetal death to asymptomatic adulthood. The onset of heart failure depends on the cross-sectional area of the tunnel and the degree of aortic insufficiency. Chronic preload due to aortic insufficiency culminates in left ventricular enlargement in asymptomatic adult patients [1–4].

## Case presentation

**Fig. 1:**
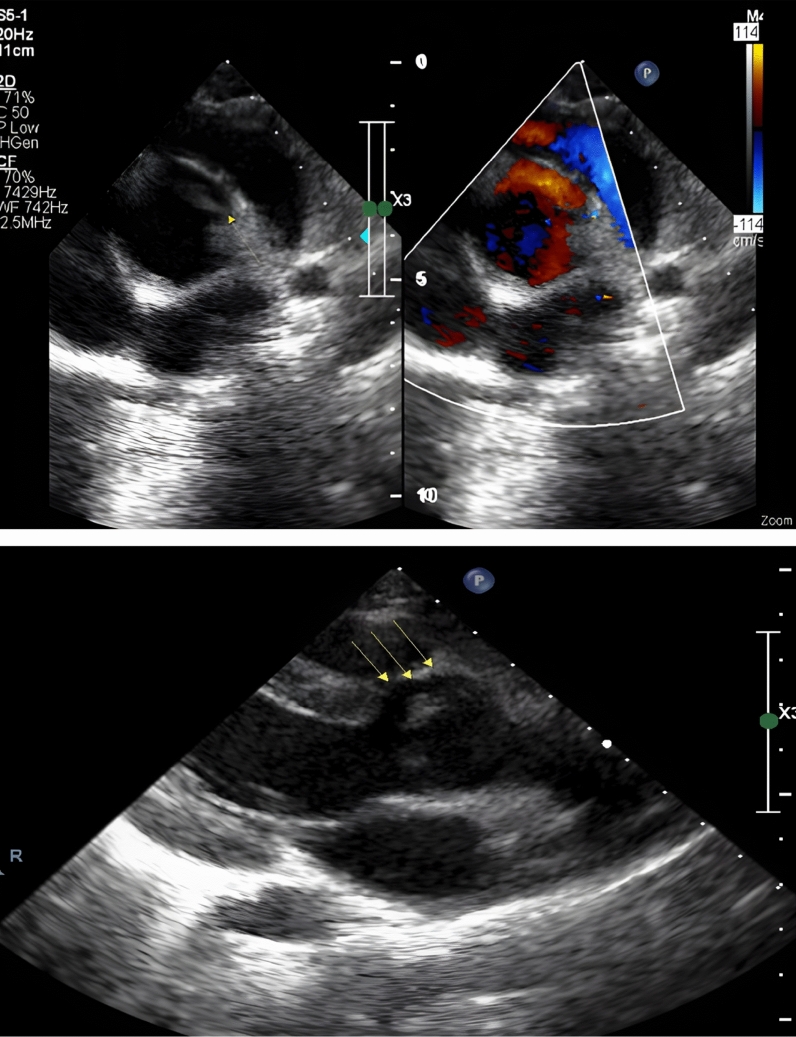
Aorto-left ventricular tunnel

**Fig. 2:**
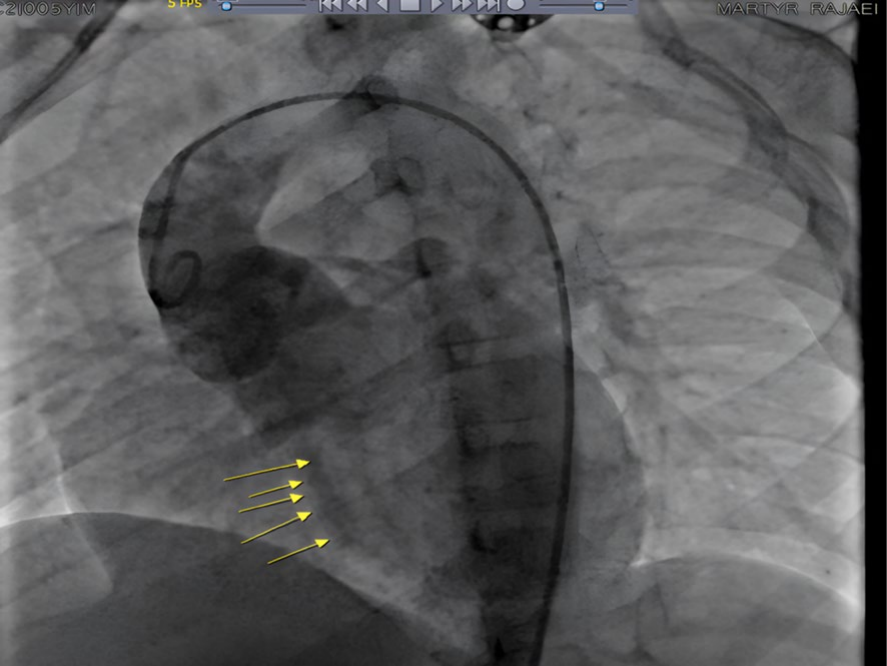
Angiogram clearly demonstrated a tunnel arising from the right aortic sinus and passing into the left ventricularcavity

A 5-year-old boy presented with fatigue during physical activity, leading to visiting a pediatric specialist. Upon examination, a continuous murmur, predominantly diastolic, was detected. Suspecting aortic insufficiency (AI), the patient was referred for further evaluation. Given the amount of color return observed, we anticipated a much lower diastolic pressure. However, the diastolic pressure was not very wide, measuring 110/65 mmHg. For this reason, we suspected a tunnel and focused on the echocardiogram this time. We observed a tunnel originating from the aorta, traversing the left ventricular septum, and opening into the left ventricular cavity. The echocardiogram report indicated an ejection fraction (EF) of 60%, severe aortic regurgitation, a velocity time integral (VTI) of 21 cm, an aortic valve ostium of 2.5 cm, and a sinus of Valsalva of 3 cm. The patient’s aortic valve was trileaflet, with a tunnel length of 11 mm and an ostium diameter of 4.5 mm (Fig. 1). After echocardiographic evaluation, we chose to use aortic root angiography rather than computed tomography angiography (CTA) because we wanted to measure hemodynamic parameters that are best assessed by catheter-based angiography, such as the degree of aortic regurgitation, left ventricular pressure, and potential coronary involvement. The angiogram clearly demonstrated a tunnel arising from the right aortic sinus and passing into the left ventricularcavity, as previously observed by echocardiography (Fig. 2).

As there was concordant evidence on both angiography and transthoracic echocardiography, the diagnosis of left ventricular–aortic tunnel was established with high certainty. Therefore, computed tomography (CT) was not warranted. The entry point of the tunnel into the right aortic sinus was located between the right coronary ostium and the right–left commissure. Therefore, the patient underwent open-heart surgery under general anesthesia with a median sternotomy. The patient was cooled to 28 °C under cardiopulmonary bypass and an aortotomy was performed. The aortic tunnel ostium was closed with an autologous pericardial patch. The tunnel ostium originated from the left and right commissures and the L and M ostium, extending obliquely through the interventricular septum into the left ventricle. Resection of fibrotic elements, sinuses, subvalvular tissue, and the right coronary cusp (RCC) and left coronary cusp (LCC) leaflets was performed. The aortic commissure was reconstructed, and the patient was successfully weaned off the pump and discharged from the hospital after7 days.

## Discussion

A tunnel between the ascending aorta and the left ventricle is a rare congenital heart disease more commonly observed in males and located above the coronary sinus. In our case, although no specific cause for this disease has been identified, various etiologies have been proposed, ranging from congenital to acquired. However, prenatal diagnosis has ruled out the acquired theory. Proximal coronary anomalies, such as an intra-tunnel coronary ostium or coronary ostial atresia, have been documented. Additionally, aortic valve anomalies, including bicuspid aortic valve and dysplasia, have been reported in previous case studies. Pulmonary valve stenosis with subvalvular pulmonary stenosis due to a tunnel has also been reported [1]. ALVT results in steal from the aorta to the ventricle and continuous murmur during diastole without impacting systemic circulation during systole. This is because during systole the left ventricle contracts and generates pressure exceeding that of the aorta, making systolic flow across the tunnel unlikely and thus not producing a murmur [5]. However, none of these conditions except for the murmur were found in our case. Regarding the diagnosis of this disease, various methods have been mentioned, including chest X-ray, transesophageal echocardiography (TEE), echocardiography, and magnetic resonance imaging (MRI). Previous studies have suggested that a high degree of aortic regurgitation in newborns may indicate the possible presence of this tunnel. In our study, this symptom was also present and the child had severe aortic regurgitation. Regarding the treatment of this condition, it has been stated that surgical intervention is recommended upon diagnosis, as delaying the procedure may lead to deterioration in the patient’s clinical condition. Two surgical methods for tunnel closure, using sutures or a patch, have been previously reported. The former, suture closure, has been associated with aortic valve distortion. In our case, we opted for the patch closure technique. Previous studies have reported cases of ALVT closure using device closure, but in our case, given the tunnel’s entry point in the right aortic sinus, specifically between the right coronary ostium and left and right aortic commissures, device closure posed significant risks [6]. In a study conducted over 11 years at a medical center involving 7 patients diagnosed with ALVT, results revealed that two patients died during follow-up, four underwent surgical tunnel closure, and one patient underwent a Bentall procedure. In this study, it was reported that 5 out of 7 patients were female. Among these, five cases were type 2, one case was type 3, and one case was type 4. Of this number, 6 cases demonstrated a tunnel ostium located above the right coronary artery, and one case showed an ostium located above the non-coronary cusp. In our case as well, the tunnel ostium was located above the right coronary artery. Aortic regurgitation was reported in only two cases from this group, both in older individuals (aged 11 and 45 years), whereas in our case, despite the patient being only 5 years old, severe aortic regurgitation was reported. This disease is classified into four types: Type 1 (a slit-like ostium at the aortic root without valve distortion), Type 2 (large extracardiac aneurysm), Type 3 (intracardiac aneurysm of the septal tunnel, with or without right ventricular outflow tract obstruction), and Type 4 (a combination of Types 2 and 3). Based on previous studies, most cases were of Type 2, whereas in our case, it was Type 3 [1]. This is significant because, in Type 1, given the anatomical conditions, it is possible to close the tunnel using a device [2]. Spontaneous closure of ALVT has been observed only in very rare, asymptomatic patients with a small tunnel. Surgery should be performed early in life and the technique chosen should be such that it stabilizes the aortic annulus without deformity and also closes the aorto-ventricular window (7).

## Conclusion

Although congenital ALVT is rare, its clinical symptoms can resemble other congenital heart diseases like TF (without pulmonary stenosis) and PDA. Differentiating features, such as the absence of wide pulse pressure, aid in distinguishing them.

## Data Availability

No datasets were generated or analysed during the current study.

## Abbreviations

ALVT: Aorto-left ventricular tunnel
TF: Tetralogy of Fallot
PDA: Patent ductus arteriosus
